# Supplementary material for: Integrated in vivo and transcriptomic analyses of lethal Oropouche virus infection reveal suppression of pathogenic host responses by antiviral therapy
Source: PLoS Pathog. 2026 Oct 7;22(10):e1014647. doi: 10.1371/journal.ppat.1014647 (PMC13657405; doi:10.1371/journal.ppat.1014647)
Supplement: Supplementary file 1 [file ppat.1014647.s007.pdf]

Gene name	Primers sequences (5' to 3')	Reference
OROVS-F	TCCGGAGGCAGCATATGTG	[1]
OROVS-R	ACAACACCAGCATTGAGCACTT	
OROVS-Probe	(FAM)-CATTTGAAGCTAGATACGG-MGB(NFQ)	
Actb -F	GTGCTATGTTGCCCTGGACT	[2]
Actb -R	GCTCGTTGCCAATGGTGATG	
CXCL10 -F	GCCATTCATCCACAGTTGACA	[3]
CXCL10 -R	CATGGTGCTGACAGTGGAGTCT	
IL-6 - F	CCTGAAAGCACTTGAAGAATTCC	[3]
IL-6 - R	GGTATGCTAAGGCACAGCACACT	
IL-10 - F	GTTGCCAAACCTTATCAGAAATGA	[4]
IL-10 - R	TTCTGGCCCGTGGTTCTCT	
ISG15 - F	AAAGCCTACAGCCATGACCT	[5]
ISG15 - R	TTAGTCAGGGGCACCAGGAA	
RSAD2 -F	CGTGAGCATCGTGAGCAATG	[6]
RSAD2 -R	TGCACCACTTCCTCAGCTTT	
TNF-α - F	GGAGTGGCTGAGCCATCGT	[3]
TNF-α - R	AGCTGGTTGTCTTTGAGAGACATG	

S1 Table. List of primers used in this study. MGB = minor-groove-binding; NFQ=non-fluorescent quencher

[1] Naveca FG, do Nascimento VA, de Souza VC, Nunes BTD, Rodrigues DSG, da Costa Vasconcelos PF. Multiplexed reverse transcription real-time polymerase chain reaction for simultaneous detection of Mayaro, Oropouche and Oropouche-like viruses. *Mem Inst Oswaldo Cruz*. 2017;112(7). doi:10.1590/0074-02760170017.

[2] Miao J, Wang JY, Robl N, Guo HR, Song SH, Peng Y, Wang YH, Huang SH, Li XJ. Validation of stable housekeeping genes for quantitative real-time PCR in golden Syrian hamster. *Indian J Anim Res*. 2021;55(10):1127–1131. doi:10.18805/IJAR.B-1237.

[3] Marzi A, Banadyga L, Haddock E, Thomas T, Shen K, Horne EJ, et al. A hamster model for Marburg virus infection accurately recapitulates Marburg hemorrhagic fever. *Sci Rep*. 2016;6: 39214. doi:10.1038/srep39214

[4] Atkins C, Miao J, Kalveram B, Juelich T, Smith JK, Perez D, et al. Natural history and pathogenesis of wild-type Marburg virus infection in STAT2 knockout hamsters. *J Infect Dis.* 2018;218:S438–S447. doi:10.1093/infdis/jiy457.

[5] Bessière P, Wasniewski M, Picard-Meyer E, Servat A, Figueroa T, et al. Intranasal type I interferon treatment is beneficial only when administered before clinical signs onset in the SARS-CoV-2 hamster model. *PLoS Pathog.* 2021;17(8):e1009427. doi:10.1371/journal.ppat.1009427.

[6] Hou L, Wu Z, Zeng P, Yang X, Shi Y, Guo J, Song J, Liu J. RSAD2 suppresses viral replication by interacting with the Senecavirus A 2C protein. *Vet Res.* 2024;55(1):115. doi:10.1186/s13567-024-01370-2.
