# Supplementary material for: Integrated in vivo and transcriptomic analyses of lethal Oropouche virus infection reveal suppression of pathogenic host responses by antiviral therapy
Source: PLoS Pathog. 2026 Oct 7;22(10):e1014647. doi: 10.1371/journal.ppat.1014647 (PMC13657405; doi:10.1371/journal.ppat.1014647)
Supplement: Supplementary file 2 [file ppat.1014647.s008.pdf]

Group	Sample ID	RNA Integrity number (RIN)/TapeStation	Reads	Yield(Gbases)	Q30 (%)
Mock (Liver)	LM1	8.6	19,539,342	5.8618	94.02
	LM2	8.8	19,238,777	5.77163	93.76
	LM3	8.8	19,153,207	5.74596	93.82
Vehicle (Liver)	LV1	8.5	32,647,818	9.79435	94.61
	LV2	8.7	44,821,940	13.44658	94.52
	LV3	8.5	44,531,729	13.35952	94.56
Treatment (Liver)	LT1	8.8	19,830,676	5.9492	94.03
	LT2	7.8	18,192,060	5.45762	93.72
	LT3	8.3	20,356,726	6.10702	93.70
Mock (Liver)	BM1	8.8	35,577,954	10.67339	97.38
	BM2	8.8	26,837,991	8.0514	93.13
	BM3	8.3	18,362,293	5.50869	93.39
Vehicle (Liver)	BV1	8.2	31,954,024	9.58621	94.05
	BV2	8.2	22,236,817	6.67105	93.48
	BV3	8.4	33,539,832	10.06195	94.28
Treatment (Liver)	BT1	8.5	19,866,906	5.96007	93.78
	BT2	8.7	20,131,326	6.0394	95.11
	BT3	8.4	20,294,218	6.08827	93.58

S2 Table. Summary of quality of isolated RNA and raw data statistics of the next generation sequencing related to Fig. 4.
