# Supplementary material for: Integrated in vivo and transcriptomic analyses of lethal Oropouche virus infection reveal suppression of pathogenic host responses by antiviral therapy
Source: PLoS Pathog. 2026 Oct 7;22(10):e1014647. doi: 10.1371/journal.ppat.1014647 (PMC13657405; doi:10.1371/journal.ppat.1014647)
Supplement: Supplementary file 3 [file ppat.1014647.s010.docx]

**Response to reviewers**

Thank you for the thoughtful and constructive feedback regarding our manuscript. The revised version of the manuscript is attached with highlighting and line numbers.

**Reviewers' Comments:**

Reviewer's Responses to Questions

**Part I - Summary**

**Reviewer #1**: This study investigates the virological, pathological, and transcriptomic features of lethal Oropouche virus (OROV) infection in the Syrian hamster model and evaluates the therapeutic efficacy of the antivirals Favipiravir and Ribavirin. The manuscript combines antiviral efficacy analyses with tissue-resolved transcriptomic profiling to better understand OROV pathogenesis and host responses.

The authors present a comprehensive virological and transcriptomic characterization of lethal OROV infection in the Syrian hamster model, while also evaluating the therapeutic efficacy of favipiravir and ribavirin. The study demonstrates that favipiravir provides robust protection against lethal disease, markedly suppressing systemic viral replication and preventing neuroinvasion, including under delayed treatment conditions. In contrast, ribavirin showed substantially lower efficacy. Importantly, the manuscript also provides what appears to be the first in vivo tissue-resolved transcriptomic framework for OROV infection, linking viral control with attenuation of inflammatory, interferon-associated, and metabolic dysregulation pathways.

Overall, the study is timely, well executed, and highly relevant given the increasing emergence and geographic expansion of OROV infections in Central and South America. The antiviral efficacy data are convincing and support the potential repurposing of favipiravir as a therapeutic candidate for OROV infection. In addition, the transcriptomic analyses substantially advance the mechanistic understanding of OROV pathogenesis and tissue-specific host responses.

The manuscript is generally well organized and clearly written. The experimental design is sound, and the integration of virological and transcriptomic datasets represents a significant strength. However, some aspects would benefit from clarification or additional supporting data to further strengthen the conclusions and improve reproducibility. In particular, additional clinical characterization and limited validation of transcriptomic findings at the protein level would substantially reinforce the mechanistic interpretation of the study.

We sincerely thank the reviewer for the thoughtful and positive evaluation of our study and for recognizing the novelty and significance of the tissue-resolved transcriptomic analysis and antiviral efficacy data. We particularly appreciate the reviewer’s comments regarding the integration of virological, pathological, and transcriptomic analyses in the Syrian hamster model.

In response to the reviewer’s suggestions, we have further strengthened the revised manuscript in several areas. First, we expanded the characterization of clinical disease progression to provide a more detailed assessment of the clinical manifestations associated with OROV infection and treatment-mediated protection. Second, we added protein-level validation of selected transcriptomic findings by Western blot, including validation of RSAD2 expression, providing additional support for the interferon-associated antiviral responses identified at the transcriptional level. We have also clarified several methodological and analytical aspects throughout the manuscript and expanded the Discussion to better address the limitations and broader significance of our findings.

We believe that these revisions have strengthened the overall characterization of OROV pathogenesis and the therapeutic effects of favipiravir, while providing a more comprehensive and balanced interpretation of the transcriptomic findings.

**Reviewer #2**: The authors of this manuscript present a timely study describing the potential utility of using the nucleoside analogue favipiravir as a novel therapy to treat Oropouche virus (OROV), which is of high interest given its ongoing emergence in the Americas. The study is well designed and executed and the manuscript written and presented in a clear and thoughtful way. The comparison between favipiravir and ribavirin provides a useful benchmark and emphasizes the potential higher utility of favipiravir given its greater efficacy in the hamster model. Importantly, the authors performed time-of-addition studies to demonstrate that the drug is also effective 48 h after infection, which is promising. The exploration of gene expression changes induced by OROV infection in the presence and absence of drug treatment underscores not only the ability of favipiravir to suppress viral replication and pathological transcriptional changes, but also provides some interesting insights into the transcriptional changes induced by OROV infection in vivo.

There are few other studies of OROV infection in vivo, and similarly few studies describing potential drugs efficacious against this virus. Therefore, this manuscript represents an important and novel contribution to the field. The study is complete and scientifically and technically rigorous. I have only three comments of a relatively minor nature.

We sincerely thank the reviewer for the careful evaluation of our manuscript and for recognizing the novelty, scientific rigor, and potential significance of our findings. We appreciate the reviewer’s positive assessment of the therapeutic efficacy of favipiravir, the delayed-treatment experiments, and the tissue-specific transcriptomic analyses.

We have carefully considered all three comments raised by the reviewer and have revised the manuscript accordingly. Specifically, we have clarified the interpretation and limitations of the temporal RT-qPCR data, expanded the discussion of the limitations associated with the use of mouse orthologues for hamster transcriptomic analysis, and further contextualized our transcriptomic findings in relation to previous studies in animal models and human OROV infection. We believe these revisions have improved the clarity and overall interpretation of the manuscript.

**Part II – Major Issues: Key Experiments Required for Acceptance**

**Reviewer #1**: The following points represent the major issues that should be addressed to strengthen the conclusions of the study and improve the overall rigor of the manuscript:

- Expanded clinical scoring and disease characterization

The manuscript would benefit from inclusion of a more detailed clinical scoring dataset. The authors describe humane endpoints and mention clinical manifestations such as lethargy and paralysis in partially protected groups; however, presentation of these data in a supplementary figure or table would improve characterization of disease progression and better distinguish lethal versus protected phenotypes. Because the study emphasizes protection from severe disease and neuroinvasion, a clearer presentation of longitudinal clinical observations would substantially strengthen the in vivo characterization of the model.

We thank the reviewer for this valuable suggestion. In response, we have expanded our evaluation of clinical disease progression in the Syrian hamster model. We have now included a detailed assessment of the major clinical signs observed throughout the course of infection and compared the disease progression among the different treatment groups. These additional data provide a clearer characterization of the clinical phenotype associated with lethal infection and treatment-mediated protection, including the progression of neurological signs in partially protected animals. The corresponding results have been incorporated into the revised manuscript and are presented in S2 Fig., as well as in the Material and Methods (Lines 410-416) and Results section (Lines 161-162).

- Validation of transcriptomic findings at the protein level

The conclusions regarding host inflammatory and antiviral responses currently rely predominantly on RNA-seq and RT-qPCR data. While the transcriptional analyses are compelling, at least limited validation at the protein level would substantially strengthen the biological interpretation of the findings. For example, quantification of selected cytokines/chemokines by ELISA or validation of representative interferon-stimulated genes by Western blot or immunohistochemistry would provide important confirmation that the observed transcriptional changes are functionally reflected at the protein level.

We thank the reviewer for this important suggestion. To provide protein-level validation of our transcriptomic findings, we performed Western blot analysis of selected interferon-stimulated genes. Although we attempted to evaluate additional targets, this analysis was technically limited by the availability of antibodies with confirmed cross-reactivity to hamster proteins. Nevertheless, we successfully validated the protein expression of Viperin encoded by *RSAD2*, a representative interferon-stimulated gene upregulated during OROV infection. These results provide additional support for the activation of IFN-associated antiviral responses during OROV infection. The corresponding data have been added to the Materials and Methods and Results sections of the revised manuscript (S6 Fig.; Lines 467-480 and 227-232).

**Reviewer #2**: None.

**Part III – Minor Issues: Editorial and Data Presentation Modifications**

**Reviewer #1**: The following comments are intended to improve methodological transparency, contextualization, and overall clarity of the manuscript. These suggestions would enhance reproducibility and strengthen the discussion of the findings:

- Clarification of treatment regimen and dosing procedures

Aiming the improvement of reproducibility and methodological transparency, please specify the exact dosing interval in hours rather than only stating “twice daily”; clarify whether animals were weighed before each administration for dose adjustment or whether dosing was based on initial body weight estimates. Ensure that the multiplicity of infection (MOI) is consistently reported across all relevant experimental descriptions.

We thank the reviewer for this helpful suggestion. We have revised the Materials and Methods section to specify the exact interval between drug administrations and to clarify the procedure for body-weight-based dose adjustments (Lines 406-408). We have also carefully reviewed the manuscript to ensure that the MOI is reported consistently across all relevant experimental descriptions.

- Figure legends and replicate information

Please clarify throughout the manuscript whether figures represent a single representative experiment or pooled data from independent experiments. In addition, the number of biological replicates, technical replicates, and individual animals should be explicitly stated in all figure legends.

We thank the reviewer for pointing this out. To improve the clarity and reproducibility of our study, we have added detailed information regarding the experimental design and replication. The revised figure legends now specify, where applicable, whether the data represent a single experiment or pooled data from independent experiments, as well as the number of biological replicates, technical replicates, and individual animals included in each analysis.

- Transcriptomic interpretation and metabolic pathways

The manuscript reports simultaneous enrichment of pathways associated with both positive regulation of innate immune responses and negative regulation of defense responses in animals with elevated viral burden. A deeper discussion of the biological significance of this apparent dual signaling profile would improve the mechanistic interpretation of the findings. In particular, the authors should better integrate these observations with the reported downregulation of metabolic and mitochondrial pathways in the liver, as this relationship may provide additional insight into OROV-associated tissue injury and systemic disease progression.

We thank the reviewer for this insightful suggestion. We have expanded the Discussion to further address the coexistence of activated innate immune responses and negatively regulated defense-related pathways in animals with higher viral burden. We have also integrated these findings with the observed downregulation of metabolic and mitochondrial pathways in the liver and discussed how sustained antiviral and inflammatory responses together with altered cellular metabolism may contribute to tissue injury and systemic disease progression during OROV infection. This additional interpretation has been incorporated into the revised Discussion (Lines 277–300; References 38-32).

- Contextualization of novelty relative to existing OROV models

Although the manuscript acknowledges prior animal models of OROV infection, the specific conceptual and technical advances of the present study should be more explicitly defined relative to the existing literature, including studies such as Rodrigues et al. (2011) or others.
Clearly distinguishing the specific advances of the present work — particularly the tissue-resolved transcriptomic analyses and therapeutic intervention approach — would better define the novelty and contribution of the study. Also, given the translational implications of favipiravir treatment, the discussion should briefly acknowledge known limitations and safety considerations associated with favipiravir use, including teratogenicity and hyperuricemia, to provide a balanced perspective on its clinical potential.

We thank the reviewer for this important suggestion. We have revised the Discussion to more clearly contextualize the conceptual and technical advances of the present study in relation to previously described OROV animal models, including Rodrigues et al. (2011). In particular, we now emphasize the combination of tissue-resolved transcriptomic profiling and therapeutic intervention in the Syrian hamster model, which provides complementary insights into OROV pathogenesis and antiviral efficacy. We have also expanded the discussion of the translational potential of favipiravir to acknowledge important limitations and safety considerations associated with its use, including its known teratogenic potential and the risk of hyperuricemia. These additions provide a more balanced assessment of the potential clinical application of favipiravir for OROV infection (Lines 301-314 and 333–341; References 35-36).

- Potential cellular characterization of inflammatory responses

If feasible, inclusion of flow cytometry or immunophenotyping data characterizing immune cell recruitment in infected tissues would provide valuable cellular context for the transcriptomic inflammatory signatures described in the manuscript. While this is not essential for acceptance, it would further strengthen the interpretation of the host-response data.

We thank the reviewer for this valuable suggestion. We agree that cellular characterization of immune cell recruitment would provide additional context for the inflammatory signatures identified by transcriptomic analysis. However, we did not perform flow cytometry or immunophenotyping, as these analyses were outside the scope of the present study. We therefore interpret the immune cell recruitment signatures identified by transcriptomic analysis as indicative of potential changes in immune cell composition or recruitment, rather than as direct evidence of cellular infiltration.

**Reviewer #2**:

**(1)** The temporal RT-qPCR data presented in Figure 5 is difficult to interpret because the infected (untreated) control is only included on day 2, and not days 6 and 8 post-infection. Clearly, this limitation is due to the untreated infected control animals not surviving to 6/8 dpi, and I cannot see a good way to overcome this limitation experimentally. However, this limitation should be acknowledged and discussed more rigorously because there is no way to know what kind of gene expression levels would be expected in infected animals later in infection, which makes the gene expression levels in drug-treated infected animals difficult to interpret.

We thank the reviewer for this thoughtful consideration of the temporal RT-qPCR data. We agree that the absence of untreated infected animals at 6 and 8 dpi represents an important limitation for interpreting the longitudinal gene expression profiles. We have revised the Discussion (Lines 322–327) to explicitly state that direct comparisons between vehicle-treated and favipiravir-treated animals at these time points were not possible and to clarify the interpretation of the observed gene expression profiles.

**(2)** In the Materials and Methods, the authors state that due to genome annotation limitations, only mouse orthologues of hamster genes were included in the RNA-Seq analysis. Again, this is a technical limitation that is difficult to overcome, but should be discussed in the relevant sections in the Results and Discussion as well to ensure that this limitation is clear to readers of the manuscript.
We thank the reviewer for highlighting this important technical limitation. As described in the Materials and Methods, the limited availability of annotated Syrian hamster genes required us to use mouse orthologues for the RNA-seq analyses. We agree that this limitation should be made more explicit beyond the Methods section. Accordingly, we have added clarification in the Results (Lines 192-194) and Discussion (Lines 315-319) describing the use of mouse orthologues and discussing the potential implications of this approach for gene annotation and pathway interpretation. These revisions make the analytical limitation more transparent to readers.

**(3)** The transcriptomic changes observed in this animal model could be more extensively compared to other studies from other animal models and humans so that the reader can place the work more completely within the wider context.

We thank the reviewer for this constructive suggestion. We have expanded the Discussion to place the transcriptomic findings observed in the Syrian hamster model within the broader context of OROV infection studies, including findings reported in other animal models and, where available, human infection. We have highlighted both the similarities and differences in host responses across experimental systems and discussed how the tissue-specific transcriptional responses identified in our study contribute to the current understanding of OROV pathogenesis. We have also emphasized the novel aspects of our tissue-resolved transcriptomic analysis and its potential relevance for understanding host responses to OROV infection (Lines 305–314).
