# Supplementary material for: Integrated in vivo and transcriptomic analyses of lethal Oropouche virus infection reveal suppression of pathogenic host responses by antiviral therapy
Source: PLoS Pathog. 2026 Oct 7;22(10):e1014647. doi: 10.1371/journal.ppat.1014647 (PMC13657405; doi:10.1371/journal.ppat.1014647)
Supplement: Peer review history

# Peer review: Decision Letter 0

**DOI:** 10.1371/journal.ppat.1014647.r001

**Authors:** Thomas Hoenen, David Safronetz

21 May 2026

PPATHOGENS-D-26-00928

Integrated in vivo and transcriptomic analyses of lethal Oropouche virus infection reveal suppression of pathogenic host responses by antiviral therapy

PLOS Pathogens

Dear Dr. Itakura,

Thank you for submitting your manuscript to PLOS Pathogens. After careful consideration, we feel that it has merit but does not fully meet PLOS Pathogens's publication criteria as it currently stands. Therefore, we invite you to submit a revised version of the manuscript that addresses the points raised during the review process.

Please submit your revised manuscript by Jul 20 2026 11:59PM. If you will need more time than this to complete your revisions, please reply to this message or contact the journal office at plospathogens@plos.org. When you're ready to submit your revision, log on to https://www.editorialmanager.com/ppathogens/ and select the 'Submissions Needing Revision' folder to locate your manuscript file.

Please include the following items when submitting your revised manuscript:

* A letter that responds to each point raised by the editor and reviewer(s). You should upload this letter as a separate file labeled 'Response to Reviewers'. This file does not need to include responses to any formatting updates and technical items listed in the 'Journal Requirements' section below.

* A marked-up copy of your manuscript that highlights changes made to the original version. You should upload this as a separate file labeled 'Revised Manuscript with Track Changes'.

* An unmarked version of your revised paper without tracked changes. You should upload this as a separate file labeled 'Manuscript'.

If you would like to make changes to your financial disclosure, competing interests statement, or data availability statement, please make these updates within the submission form at the time of resubmission. Guidelines for resubmitting your figure files are available below the reviewer comments at the end of this letter. As the corresponding author, your ORCID iD is verified in the submission system and will appear in the published article. PLOS supports the use of ORCID, and we encourage all coauthors to register for an ORCID iD and use it as well. Please encourage your coauthors to verify their ORCID iD within the submission system before final acceptance, as unverified ORCID iDs will not appear in the published article. Only the individual author can complete the verification step; PLOS staff cannot verify ORCID iDs on behalf of authors.

We look forward to receiving your revised manuscript.

Kind regards,

David Safronetz, Ph.D.

Academic Editor

PLOS Pathogens

Thomas Hoenen

Section Editor

PLOS PathogensSumita Bhaduri-McIntosh

Editor-in-Chief

PLOS Pathogens

orcid.org/0000-0003-2946-9497

Michael Malim

Editor-in-Chief

PLOS Pathogens

orcid.org/0000-0002-7699-2064

**Journal Requirements:**

If the reviewer comments include a recommendation to cite specific previously published works, please review and evaluate these publications to determine whether they are relevant and should be cited. There is no requirement to cite these works unless the editor has indicated otherwise.

1) Please ensure that the CRediT author contributions listed for every co-author are completed accurately and in full.

At this stage, the following Authors/Authors require contributions: Cássia Sousa Moraes, Gabriel Gonzalez, Akihiko Sato, Shigeru Miki, Atsuko Inoue, Koshiro Tabata, Joshua W. Kranrod, Chilekwa Frances Kabamba, Aiko Ohnuma, Keita Matsuno, Rio Harada, Shinji Saito, Michihito Sasaki, Yasuko Orba, William W. Hall, Hirofumi Sawa, and Yukari Itakura. Please ensure that the full contributions of each author are acknowledged in the "Add/Edit/Remove Authors" section of our submission form.

The list of CRediT author contributions may be found here: https://journals.plos.org/plospathogens/s/authorship#loc-author-contributions

2) Please provide an Author Summary. This should appear in your manuscript between the Abstract (if applicable) and the Introduction, and should be 150-200 words long. The aim should be to make your findings accessible to a wide audience that includes both scientists and non-scientists. Sample summaries can be found on our website under Submission Guidelines:

https://journals.plos.org/plospathogens/s/submission-guidelines#loc-parts-of-a-submission

3) Please upload all main figures as separate Figure files [Figures 1-5] in .tif or .eps format. For more information about how to convert and format your figure files please see our guidelines:

https://journals.plos.org/plospathogens/s/figures

4) We have noticed that you have uploaded Supporting Information files [Supplementary figures 4 and supplementary tables 2], but you have not included a list of legends. Please add a full list of legends for your Supporting Information files after the references list.

5) Please send a completed 'Competing Interests' statement, including any COIs declared by your co-authors. If you have no competing interests to declare, please state "The authors have declared that no competing interests exist". Otherwise please declare all competing interests beginning with the statement "I have read the journal's policy and the authors of this manuscript have the following competing interests:"

6) Thank you for stating financial disclosure statement. Please include the following:

i) State what role the funders took in the study. If the funders had no role in your study, please state: “The funders had no role in study design, data collection and analysis, decision to publish, or preparation of the manuscript.”    ii) . If any authors received a salary from any of your funders, please state which authors and which funders.

7) Your current Financial Disclosure states, "This study was supported in part by the Japan Society for the Promotion of Science (JSPS) KAKENHI under grant numbers 25K18810 and 25K09431; and the Japan Agency for Medical Research and Development (AMED) under grant numbers JP223fa627005 and JP24wm0225044". However, your funding information on the submission form is missing Grant numbers "25K09431 and JP24wm0225044." Please indicate by return email the full and correct funding information for your study and confirm the order in which funding contributions should appear. Please be sure to indicate whether the funders played any role in the study design, data collection and analysis, decision to publish, or preparation of the manuscript.

**Reviewers' Comments:**

Reviewer's Responses to Questions

**Part I - Summary**

Please use this section to discuss strengths/weaknesses of study, novelty/significance, general execution and scholarship.

Reviewer #1: This study investigates the virological, pathological, and transcriptomic features of lethal Oropouche virus (OROV) infection in the Syrian hamster model and evaluates the therapeutic efficacy of the antivirals Favipiravir and Ribavirin. The manuscript combines antiviral efficacy analyses with tissue-resolved transcriptomic profiling to better understand OROV pathogenesis and host responses.

The authors present a comprehensive virological and transcriptomic characterization of lethal OROV infection in the Syrian hamster model, while also evaluating the therapeutic efficacy of favipiravir and ribavirin. The study demonstrates that favipiravir provides robust protection against lethal disease, markedly suppressing systemic viral replication and preventing neuroinvasion, including under delayed treatment conditions. In contrast, ribavirin showed substantially lower efficacy. Importantly, the manuscript also provides what appears to be the first in vivo tissue-resolved transcriptomic framework for OROV infection, linking viral control with attenuation of inflammatory, interferon-associated, and metabolic dysregulation pathways.

Overall, the study is timely, well executed, and highly relevant given the increasing emergence and geographic expansion of OROV infections in Central and South America. The antiviral efficacy data are convincing and support the potential repurposing of favipiravir as a therapeutic candidate for OROV infection. In addition, the transcriptomic analyses substantially advance the mechanistic understanding of OROV pathogenesis and tissue-specific host responses.

The manuscript is generally well organized and clearly written. The experimental design is sound, and the integration of virological and transcriptomic datasets represents a significant strength. However, some aspects would benefit from clarification or additional supporting data to further strengthen the conclusions and improve reproducibility. In particular, additional clinical characterization and limited validation of transcriptomic findings at the protein level would substantially reinforce the mechanistic interpretation of the study.

Reviewer #2: The authors of this manuscript present a timely study describing the potential utility of using the nucleoside analogue favipiravir as a novel therapy to treat Oropouche virus (OROV), which is of high interest given its ongoing emergence in the Americas. The study is well designed and executed and the manuscript written and presented in a clear and thoughtful way. The comparison between favipiravir and ribavirin provides a useful benchmark and emphasizes the potential higher utility of favipiravir given its greater efficacy in the hamster model. Importantly, the authors performed time-of-addition studies to demonstrate that the drug is also effective 48 h after infection, which is promising. The exploration of gene expression changes induced by OROV infection in the presence and absence of drug treatment underscores not only the ability of favipiravir to suppress viral replication and pathological transcriptional changes, but also provides some interesting insights into the transcriptional changes induced by OROV infection in vivo.

There are few other studies of OROV infection in vivo, and similarly few studies describing potential drugs efficacious against this virus. Therefore, this manuscript represents an important and novel contribution to the field. The study is complete and scientifically and technically rigorous. I have only three comments of a relatively minor nature.

**********

**Part II – Major Issues: Key Experiments Required for Acceptance**

Please use this section to detail the key new experiments or modifications of existing experiments that should be absolutely required to validate study conclusions.

Generally, there should be no more than 3 such required experiments or major modifications for a "Major Revision" recommendation. If more than 3 experiments are necessary to validate the study conclusions, then you are encouraged to recommend "Reject".

Reviewer #1: The following points represent the major issues that should be addressed to strengthen the conclusions of the study and improve the overall rigor of the manuscript:

- Expanded clinical scoring and disease characterization

The manuscript would benefit from inclusion of a more detailed clinical scoring dataset. The authors describe humane endpoints and mention clinical manifestations such as lethargy and paralysis in partially protected groups; however, presentation of these data in a supplementary figure or table would improve characterization of disease progression and better distinguish lethal versus protected phenotypes. Because the study emphasizes protection from severe disease and neuroinvasion, a clearer presentation of longitudinal clinical observations would substantially strengthen the in vivo characterization of the model.

- Validation of transcriptomic findings at the protein level

The conclusions regarding host inflammatory and antiviral responses currently rely predominantly on RNA-seq and RT-qPCR data. While the transcriptional analyses are compelling, at least limited validation at the protein level would substantially strengthen the biological interpretation of the findings. For example, quantification of selected cytokines/chemokines by ELISA or validation of representative interferon-stimulated genes by Western blot or immunohistochemistry would provide important confirmation that the observed transcriptional changes are functionally reflected at the protein level.

Reviewer #2: None.

**********

**Part III – Minor Issues: Editorial and Data Presentation Modifications**

Please use this section for editorial suggestions as well as relatively minor modifications of existing data that would enhance clarity.

Reviewer #1: The following comments are intended to improve methodological transparency, contextualization, and overall clarity of the manuscript. These suggestions would enhance reproducibility and strengthen the discussion of the findings:

- Clarification of treatment regimen and dosing procedures

Aiming the improvement of reproducibility and methodological transparency, please specify the exact dosing interval in hours rather than only stating “twice daily”; clarify whether animals were weighed before each administration for dose adjustment or whether dosing was based on initial body weight estimates. Ensure that the multiplicity of infection (MOI) is consistently reported across all relevant experimental descriptions.

- Figure legends and replicate information

Please clarify throughout the manuscript whether figures represent a single representative experiment or pooled data from independent experiments. In addition, the number of biological replicates, technical replicates, and individual animals should be explicitly stated in all figure legends.

- Transcriptomic interpretation and metabolic pathways

The manuscript reports simultaneous enrichment of pathways associated with both positive regulation of innate immune responses and negative regulation of defense responses in animals with elevated viral burden. A deeper discussion of the biological significance of this apparent dual signaling profile would improve the mechanistic interpretation of the findings. In particular, the authors should better integrate these observations with the reported downregulation of metabolic and mitochondrial pathways in the liver, as this relationship may provide additional insight into OROV-associated tissue injury and systemic disease progression.

- Contextualization of novelty relative to existing OROV models

Although the manuscript acknowledges prior animal models of OROV infection, the specific conceptual and technical advances of the present study should be more explicitly defined relative to the existing literature, including studies such as Rodrigues et al. (2011) or others.

Clearly distinguishing the specific advances of the present work — particularly the tissue-resolved transcriptomic analyses and therapeutic intervention approach — would better define the novelty and contribution of the study. Also, given the translational implications of favipiravir treatment, the discussion should briefly acknowledge known limitations and safety considerations associated with favipiravir use, including teratogenicity and hyperuricaemia, to provide a balanced perspective on its clinical potential.

- Potential cellular characterization of inflammatory responses

If feasible, inclusion of flow cytometry or immunophenotyping data characterizing immune cell recruitment in infected tissues would provide valuable cellular context for the transcriptomic inflammatory signatures described in the manuscript. While this is not essential for acceptance, it would further strengthen the interpretation of the host-response data.

Reviewer #2: (1) The temporal RT-qPCR data presented in Figure 5 is difficult to interpret because the infected (untreated) control is only included on day 2, and not days 6 and 8 post-infection. Clearly, this limitation is due to the untreated infected control animals not surviving to 6/8 dpi, and I cannot see a good way to overcome this limitation experimentally. However, this limitation should be acknowledged and discussed more rigorously because there is no way to know what kind of gene expression levels would be expected in infected animals later in infection, which makes the gene expression levels in drug-treated infected animals difficult to interpret.

(2) In the Materials and Methods, the authors state that due to genome annotation limitations, only mouse orthologues of hamster genes were included in the RNA-Seq analysis. Again, this is a technical limitation that is difficult to overcome, but should be discussed in the relevant sections in the Results and Discussion as well to ensure that this limitation is clear to readers of the manuscript.

(3) The transcriptomic changes observed in this animal model could be more extensively compared to other studies from other animal models and humans so that the reader can place the work more completely within the wider context.

**********

PLOS authors have the option to publish the peer review history of their article (what does this mean?). If published, this will include your full peer review and any attached files.

If you choose “no”, your identity will remain anonymous but your review may still be made public.

**Do you want your identity to be public for this peer review?**  For information about this choice, including consent withdrawal, please see our Privacy Policy.

Reviewer #1: No

Reviewer #2: No

[NOTE: If reviewer comments were submitted as an attachment file, they will be attached to this email and accessible via the submission site. Please log into your account, locate the manuscript record, and check for the action link "View Attachments". If this link does not appear, there are no attachment files.]

**Figure resubmission:**

While revising your submission, we strongly recommend that you use PLOS’s NAAS tool (https://ngplosjournals.pagemajik.ai/artanalysis) to test your figure files. NAAS can convert your figure files to the TIFF file type and meet basic requirements (such as print size, resolution), or provide you with a report on issues that do not meet our requirements and that NAAS cannot fix.

After uploading your figures to PLOS’s NAAS tool - https://ngplosjournals.pagemajik.ai/artanalysis, NAAS will process the files provided and display the results in the "Uploaded Files" section of the page as the processing is complete. If the uploaded figures meet our requirements (or NAAS is able to fix the files to meet our requirements), the figure will be marked as "fixed" above. If NAAS is unable to fix the files, a red "failed" label will appear above. When NAAS has confirmed that the figure files meet our requirements, please download the file via the download option, and include these NAAS processed figure files when submitting your revised manuscript.

**Reproducibility:**

To enhance the reproducibility of your results, we recommend that authors of applicable studies deposit laboratory protocols in protocols.io, where a protocol can be assigned its own identifier (DOI) such that it can be cited independently in the future. Additionally, PLOS ONE offers an option to publish peer-reviewed clinical study protocols. Read more information on sharing protocols at https://plos.org/protocols?utm_medium=editorial-email&utm_source=authorletters&utm_campaign=protocols

# Author comment: Author response to Decision Letter 1

**DOI:** 10.1371/journal.ppat.1014647.r002

25 Aug 2026

Attachment Submitted filename: 260820_Response to Reviewers.docx

# Peer review: Decision Letter 1

**DOI:** 10.1371/journal.ppat.1014647.r003

**Authors:** Thomas Hoenen, David Safronetz

21 Sep 2026

Dear Dr. Itakura,

We are pleased to inform that your manuscript, "Integrated in vivo and transcriptomic analyses of lethal Oropouche virus infection reveal suppression of pathogenic host responses by antiviral therapy", has been editorially accepted for publication at PLOS Pathogens.

Before your manuscript can be formally accepted and sent to production, you will need to complete our formatting changes, which you will receive by email within a week. Please note that your manuscript will not be scheduled for publication until you have made the required changes.

IMPORTANT NOTES

(1) Copyediting and Proofreading: The corresponding author will receive a typeset proof for review, to ensure errors have not been introduced during production. Please review the PDF proof of your manuscript carefully, as this is the last chance to correct any errors. Please note that major changes, or those which affect the scientific understanding of the work, will likely cause delays to the publication date of your manuscript.

(2) Appropriate Figure Files: Please remove all name and figure # text from your figure files. Please also take this time to check that your figures are of high resolution, which will improve the readbility of your figures and help expedite your manuscript's publication. Please note that figures must have been originally created at 300dpi or higher. Do not manually increase the resolution of your files. For instructions on how to properly obtain high quality images, please review our Figure Guidelines, with examples at: http://journals.plos.org/plospathogens/s/figures.

We encourage you to use PACE (http://pace.apexcovantage.com/), a figure-checking tool which helps authors ensure all figures meet PLOS requirements so that figures can publish in the highest quality. PACE is a digital diagnostic and conversion tool for figure files. It will provide information about any failed check(s) and, if able, will automatically convert the figure into an acceptable file that passes quality checks.

To use PACE, you must first register as a user. Then, login and navigate to the UPLOAD tab, where you will find detailed instructions on how to use the tool.

(3) Striking Image: Please upload a striking still image to accompany your article if one is available (you can include a new image or an existing one from within your manuscript). Should your paper be accepted, this image will be considered for our monthly issue image and may also appear on our website to feature your article. Please upload this as a separate file, selecting "striking image" as the file type upon upload. Please also include a separate "Other" file with a caption, including credits and any potential copyright information. Please do not include the caption in the main article file. If your image is from someone other than yourself, please ensure that the artist has read and agreed to the terms and conditions of the Creative Commons Attribution License at http://journals.plos.org/plospathogens/s/content-license. Please note that PLOS cannot publish copyrighted images.

(4) Press Release or Related Media: If you or your institution will be preparing press materials for this manuscript, please inform our press team in advance at plospathogens@plos.org as soon as possible. We ask that you contact us within one week to plan ahead of our fast Production schedule. If you need to know your paper's publication date for related media purposes, you must coordinate with our press team, and your manuscript will remain under a strict press embargo until the publication date and time.

(5) Update your Profile Information: Now that your manuscript has been provisionally accepted, please log into Editorial Manager and update your profile, if needed. Go to http://www.editorialmanager.com/ppathogens, log in, and click on the "Update My Information" link at the top of the page. Please update your user information to ensure an efficient production and billing process.

(6) LaTeX users only: Our staff will ask you to upload a TEX file in addition to the PDF before the paper can be sent to typesetting, so please carefully review our Latex Guidelines http://journals.plos.org/plospathogens/s/latex in the meantime.

Thank you again for supporting Open Access publishing; we are looking forward to publishing your work in PLOS Pathogens.

Best regards,

David Safronetz, Ph.D.

Academic Editor

PLOS Pathogens

Thomas Hoenen

Section Editor

PLOS Pathogens

Dominique Soldati-Favre

Editor-in-Chief

PLOS Pathogens

***********************************************************

Reviewer's Responses to Questions

**Part I - Summary**

Please use this section to discuss strengths/weaknesses of study, novelty/significance, general execution and scholarship.<br/><br/>

Reviewer #1: The authors provide a manuscript with a valuable contribution to the understanding of Oropouche virus (OROV) infection and antiviral treatment in a lethal Syrian hamster model. The study evaluates the efficacy of favipiravir and ribavirin in vitro and in vivo. The tissue-resolved transcriptional profiles of host responses add an important dimension to the current understanding of OROV pathogenesis and the alterations associated with antiviral response and treatment.

The questions raised during the previous review have been clarified and appropriately addressed by the authors, with corrections incorporated where pertinent. In my view, the revisions have improved the clarity and presentation of the manuscript.

Prior to publication, I suggest considering the following minor points. In Figure S2E, the authors may wish to verify whether there is indeed an improvement in the animals' clinical scores between days 4 and 5, as suggested by the clinical scoring data. In Figure S6, it would be interesting to clarify why different favipiravir doses do not appear to produce markedly different effects on viperin expression in the liver, whereas a dose-dependent difference is more apparent in the brain, where viperin levels in animals treated with 100 mg/kg/day of favipiravir and 40 mg/kg/day of ribavirin appear comparable to those observed in infected, untreated animals.

These are minor points that do not alter my overall assessment. From my perspective, the manuscript is suitable for publication, and I recommend acceptance.

Reviewer #2: The authors have made some efforts to address my previous comments, however, the work is still not sufficiently placed within the broader literature (my previous comment 3). Most notably, there are two other transcriptomics datasets for OROV available, one that is not cited (PMID 42531721) and one that is cited but not discussed on the context of how much agreement there is between datesets (41806841). This discussion would be important given that the hamster model may not reflect human transcriptomics changes and given that there were unavoidable complications related to the limited genome annotation in this species.

There are other instances where the established literature is insufficiently discussed. Most notably, PMID 40937847 also shows clear post-infection therapeutic benefits of another drug (4'-fluorouridine) when used up to four days post-infection in mice, and that study includes multiple viral strains. I would expect to see here a more in-depth discussion of the different inflammatory and virological outcomes when comparing these different treatments. These omissions leave the reader with a rather positive spin on the benefits of favipiravir that are not sufficiently critically evaluated in the context of the established literature.

--------------------

**Part II – Major Issues: Key Experiments Required for Acceptance**

Please use this section to detail the key new experiments or modifications of existing experiments that should be absolutely required to validate study conclusions.<br/><br/>Generally, there should be no more than 3 such required experiments or major modifications for a "Major Revision" recommendation. If more than 3 experiments are necessary to validate the study conclusions, then you are encouraged to recommend "Reject".<br/><br/>

Reviewer #1: None.

Reviewer #2: (No Response)

--------------------

**Part III – Minor Issues: Editorial and Data Presentation Modifications**

Please use this section for editorial suggestions as well as relatively minor modifications of existing data that would enhance clarity.<br/><br/>

Reviewer #1: None.

Reviewer #2: (No Response)

--------------------

PLOS authors have the option to publish the peer review history of their article (what does this mean?). If published, this will include your full peer review and any attached files.

If you choose “no”, your identity will remain anonymous but your review may still be made public.

**Do you want your identity to be public for this peer review?**  For information about this choice, including consent withdrawal, please see our Privacy Policy.

Reviewer #1: No

Reviewer #2: No

# Editor report: Acceptance letter

**DOI:** 10.1371/journal.ppat.1014647.r004

**Authors:** Thomas Hoenen, David Safronetz

Dear Dr. Itakura,

We are delighted to inform you that your manuscript, "Integrated in vivo and transcriptomic analyses of lethal Oropouche virus infection reveal suppression of pathogenic host responses by antiviral therapy," has been formally accepted for publication in PLOS Pathogens.

We have now passed your article onto the PLOS Production Department who will complete the rest of the pre-publication process. All authors will receive a confirmation email upon publication.

Thank you again for supporting open-access publishing; we are looking forward to publishing your work in PLOS Pathogens.

Best regards,

Dominique Soldati-Favre

Editor-in-Chief

PLOS Pathogens
